# Smartphone Application for Smoking Cessation (Quit with US): A Randomized Controlled Trial among Young Adult Light Smokers in Thailand

**DOI:** 10.3390/ijerph19148265

**Published:** 2022-07-06

**Authors:** Phantara Chulasai, Dujrudee Chinwong, Purida Vientong, Sunee Lertsinudom, Penkarn Kanjanarat, John J. Hall, Surarong Chinwong

**Affiliations:** 1 PhD’s Degree Program in Pharmacy, Faculty of Pharmacy, Chiang Mai University, Chiang Mai 50200, Thailand; panthara.chula@gmail.com; 2 Department of Social Pharmacy, Faculty of Pharmacy, Payap University, Chiang Mai 50000, Thailand; 3 Department of Pharmaceutical Care, Faculty of Pharmacy, Chiang Mai University, Chiang Mai 50200, Thailand; dujrudee.c@cmu.ac.th (D.C.); purida.v@cmu.ac.th (P.V.); penkarn.k@cmu.ac.th (P.K.); 4 Center of Excellence for Innovation in Analytical Science and Technology for Biodiversity-Based Economic and Society (I-ANALY-S-T_B.BES-CMU), Chiang Mai University, Chiang Mai 50200, Thailand; 5 Division of Clinical Pharmacy, Faculty of Pharmaceutical Sciences, Khon Kaen University, Khon Kaen 40002, Thailand; lsunee@kku.ac.th; 6 School of Population Health, Faculty of Medicine, University of New South Wales, Sydney 2052, Australia; john.hall@unsw.edu.au

**Keywords:** smartphone application, smartphone app, smoking cessation, young adult smoker, randomized controlled trial

## Abstract

This study aimed to determine the efficacy of a smartphone application named Quit with US among young adult smokers. An open-label, parallel, 2-group, randomized controlled trial with a 12-week follow-up was conducted between March and November 2020 among undergraduate students (18 to 24 years) in Chiang Mai Province, Thailand. A total of 273 participants were assigned by simple randomization procedure to the Quit with US intervention group (*n* = 137) or the control group (*n* = 136). All participants received pharmacists’ smoking cessation counseling at baseline and follow-ups. In addition, the intervention group’s participants were advised to use Quit with US. The baseline and 12-week follow-up assessments were conducted at a study unit, whereas other follow-ups were completed over the telephone. The primary abstinence outcome was the exhaled CO concentration level (≤6 ppm) verified 7-day point prevalence abstinence. At baseline, the participants’ mean (standard deviation) age was 21.06 (1.62) years. Most identified as daily smokers (57.9%, *n* = 158), consumed ≤10 cigarettes daily (89.4%, *n* = 244), and expressed low level of nicotine dependence as measured by Heaviness of Smoking Index score (86.1%, *n* = 235). Regarding intention-to-treat analyses, participants in the Quit with US intervention group achieved significantly greater smoking abstinence rate than those in the control group (58.4% (80/137) vs. 30.9% (42/136), risk ratio = 1.89, 95% confidence intervals = 1.42 to 2.52, *p* < 0.001). In conclusion, Quit with US integrated with pharmacists’ smoking cessation counseling significantly enhanced smoking abstinence rates among young adult light smokers consuming ≤ 10 cigarettes daily.

## 1. Introduction

Smoking remains one of the leading causes of preventable deaths worldwide, including in Thailand [1,2]. Despite the positive impacts of tobacco control policies on a continual decrease in the overall prevalence of smoking, challenges remain concerning the expanding smoking habits in young adult populations [3]. In addition, young adult smokers (18 to 24 years) exhibited the lowest smoking cessation rate compared with other populations [4]. Young adult smokers were more likely to be occasional smokers and described lower cigarette consumption than adult smokers [5]. Even though they attempted to quit smoking, their success rate remained lower than that of adult smokers due to very few accessed evidence-based smoking cessation approaches [5,6,7]. Therefore, identifying an effective smoking cessation approach for young adult smokers remains necessary to assist them in smoking cessation and further decrease smoking prevalence.

Although various evidence-based smoking cessation approaches are available, effective smoking cessation approaches for young adult smokers have not yet been conclusively identified [8,9]. Community pharmacists’ smoking cessation counseling is deemed effective as suggested in various national guidelines [10,11,12]. In Thailand, although this approach yields cost savings, the scalability remains limited [13,14,15]. Related randomized controlled trials (RCTs) considering smartphone applications (apps) for smoking cessation revealed various results demonstrating both negative and positive effects on smoking cessation rate [16,17,18,19,20,21,22,23,24]. A related systematic review emphasized that smartphone apps tended to enhance success in smoking cessation in young adult populations [25]. Young adults are more likely to engage with digital technologies and are the highest ranked users of the internet using their smartphones [26].

Smartphone apps for smoking cessation combine considerable benefits and overcome barriers from other approaches. Their content is always available regardless of location. They have the ability in tailoring individualized contents and dynamically updating depending on a user’s input information. Importantly, they can be designed to appeal to specific target users [27,28]. Despite several smartphone apps for smoking cessation being available worldwide, very few adhere to evidence-based cessation approaches [29]. Moreover, none of the smartphone apps provides the whole content of the 5A’s treatment for providing smoking cessation consultation as recommended by the World Health Organization [30,31] and several countries [11,12].

Combining the benefits of smartphone apps for smoking cessation with age-appropriate technology, Quit with US, a novel smartphone app, was intentionally invented in Thailand to encourage young adult smokers (18 to 24 years) to achieve smoking cessation [32]. The invention of the smartphone app adhered to guidelines for smoking cessation treatment that particularly concentrated on the 5A’s model along with self-efficacy theory. The 5A’s model is associated with a significant increase in successful smoking cessation [33,34]. It consists of five steps to promote smoking cessation: ask, advise, assess, assist and arrange. In addition, improvement in self-efficacy, an individual’s belief in their own ability to carry out specific health behaviors, enhanced success in smoking cessation and prevented smoking relapse [35,36,37,38].

Recently, a single arm, pre-post study assessed preliminary results across a comprehensive range of outcome measures after using Quit with US for four weeks [32]. The findings demonstrated that the smartphone app possibly benefited promoting smoking cessation and improved smoking behaviors as well as knowledge and attitudes toward smoking and smoking cessation. In addition, young adult smokers demonstrated satisfaction and confidence in using it. Therefore, this RCT aimed to determine the efficacy of Quit with US among a larger group of young adult smokers.

## 2. Materials and Methods

### 2.1. Study Design and Participants

This study employed an open-label, parallel, 2-group, RCT with a 12-week follow-up. The study was registered on the Thai Clinical Trials Registry (TCTR20190205007) and was reported in accordance with CONSORT statements.

Participants were recruited simultaneously from undergraduate students from five universities in Chiang Mai Province, northern Thailand. Eligibility criteria included the following: (1) aged 18 to 24 years; (2) smoked minimally one cigarette within the previous 30 days; (3) interested in smoking cessation in the next 30 days; (4) possession of a smartphone using iOS and Android operating system supporting the smartphone’s app use; (5) able to access an internet connection, and (6) ability to communicate in Thai. Individuals meeting any of the following criteria were excluded from the study: (1) participating in other smoking cessation programs; (2) history of using other forms of tobacco apart from cigarettes in the last 30 days, and (3) history of using any other treatment for smoking cessation in the last 30 days. Participants were provided 400 THB (approximately USD 12.50) as compensation for completing the entire study.

### 2.2. Recruitment

Between March 9 and 28 August 2020, recruitment procedures were undertaken using digital advertising on Facebook and LINE app, and referrals from friends and family members. After completing a telephone-based screening, eligible participants were asked to meet with a research team at any of four study units nearby their university. The study units were organized in a consultation room of a university affiliated pharmacy of the Faculty of Pharmacy, Chiang Mai University and Payap University, and two community pharmacies. Participants were thoroughly notified of all the study’s details as described in the subject information sheet. Once the informed consent form was signed, the participant was consecutively enrolled in the study.

### 2.3. Randomization

Participants were randomly assigned with an equal probability (1:1) to either the Quit with US intervention group or to the control group. According to a simple randomization procedure, a research team generated the random allocation sequences by referring to a table of random numbers [39]. The allocation sequence was hidden in opaque sequentially numbered sealed envelopes. The researcher responsible for enrolling participants had no access to the upcoming group assignment, and randomized participants were given a unique identifier code.

### 2.4. Pharmacists’ Smoking Cessation Counseling

Both participants assigned to the Quit with US intervention and control groups received the pharmacists’ smoking cessation counseling at baseline and follow-ups. The smoking cessation counseling was provided by one experienced pharmacist in the research team who had successfully completed the National Tobacco Cessation Provider (TCP) Program and was certified by the Medical Association of Thailand. The smoking cessation counseling was implemented by applying the research counseling manual (Table S1) written to comply with national guidelines for smoking cessation treatment [12]. At baseline, the counseling included both face-to-face and group counseling and lasted between 15 and 30 min, depending upon the pharmacist and participants. Additionally, the follow-up counseling focusing on the progress in quitting smoking was provided at 1, 2, 3, 4, 6, and 8 weeks post randomization by telephone. All participants were asked to record the discussion during the pharmacist counseling process.

In addition, smoking cessation products were prepared to be provided for free to participants (as needed) in accordance with Thai smoking cessation guidelines [12]. Participants were able to send text messages or telephone the research team to receive additional support as needed. Given the nature of the study protocol, neither the participants nor the research team could be blinded as to group assignment.

### 2.5. Quit with US Intervention Group

In addition to the pharmacists’ smoking cessation counseling, participants in the intervention group had to download and install Quit with US on their smartphones. They were instructed to log on using only one username and a password generated by the research team, and use the smartphone app minimally once daily across the 12-week study.

Additional information of the smartphone apps’ design and development was described in detail elsewhere [32]. Briefly, Quit with US was intentionally invented in Thai for iOS and Android operating systems to encourage smoking cessation. The displayed information of Quit with US is particularized based on users’ smoking habits, for instance, daily cigarette consumption, time to the first cigarette after waking up and willingness to quit smoking.

Quit with US comprises five main pages. *Suggested by US* advises regarding quitting smoking by offering information on the disadvantages of smoking and offering recommendations on quitting smoking. *Talk with US* arranges a follow-up communication by displaying a list of questions regarding smoking cessation from anonymous users and pharmacists’ answers. One experienced pharmacist in the research team responded to the questions by logging in with a unique username and password. *Quit with US* assesses the reason for tobacco addiction and users’ willingness to quit smoking. Additionally, this page assists quitting smoking with a personalized quit plan by displaying users’ self-set target date and reason for quitting smoking. *Let US Help* assists quitting smoking by introducing coping methods for nicotine cravings or unintentional smoking. *Success of US* arranges a self-monitoring of quitting smoking by displaying users’ diary and users’ progress in quitting smoking.

### 2.6. Control Group

Participants assigned to the control group received only the pharmacists’ smoking cessation counseling. In addition, to avoid contamination with the intervention group, they were requested to cooperate in avoiding access to Quit with US and other smartphone apps until finishing the 12-week follow-up period.

### 2.7. Baseline and Follow-Up

As part of a baseline assessment, baseline data were collected using a self-administered questionnaire completed by all participants. A breath test using a handheld monitor, piCO Smokerlyzer [40], was then conducted to measure the concentration level of exhaled carbon monoxide (CO).

The follow-up assessment was conducted at 1, 2, 3, 4, 6, and 8 weeks post randomization by telephone. The self-reported smoking abstinence was assessed. Nonresponding participants were telephoned every other day for a maximum of three times. After that, they were considered as nonabstainers, as per intention-to-treat principle.

**Figure 1:**
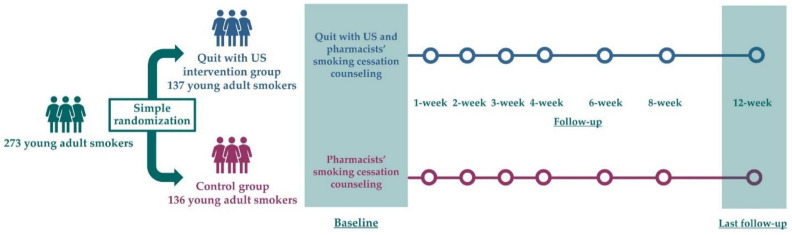
Study protocol of Quit with US Trial.

The last follow-up assessment was conducted at 12-week post randomization, between June 1 and November 23, 2020. Follow-up data and the exhaled carbon monoxide (CO) concentration level were collected from all participants in the same manner as at baseline. For nonresponding participants, three reminders with two days apart text messages followed by a maximum of three telephone calls every other day were sent. After that, they were considered as nonabstainers, as per intention-to-treat principle (Figure 1).

### 2.8. Measures

#### 2.8.1. Primary Outcome

The primary smoking abstinence outcome was 7-day point prevalence at the 12-week follow-up, as recommended for smoking abstinence measures [41]. The outcome was defined as a self-report of the previous seven consecutive days of continuous abstinence from smoking plus an exhaled CO concentration level of ≤6 parts per million (ppm) [40]. Participants lost to follow-up were assumed as nonabstainers, as per intention-to-treat principle.

#### 2.8.2. Secondary Outcomes

The secondary outcomes including change in smoking behaviors, change in exhaled CO concentration level, and change in knowledge and attitudes toward smoking and smoking cessation were assessed and compared between baseline and 12-week follow-up. In addition, use of Quit with US outcome was assessed by only participants in the intervention group at the 12-week follow-up. Information regarding the questionnaire development was described in detail elsewhere [32].

Smoking behavior measures comprised daily cigarette consumption and nicotine dependence level measured by Heaviness of Smoking Index (HSI) score. The HSI score ranged between zero and six, where high HSI score conveyed high nicotine dependence [42].

Knowledge and attitude measures each comprised 15 question items, with high aggregate scores conveying positive outcomes. Concerning knowledge measure, true or false answers were given to items; incorrect answer (zero score) or correct answer (one score). The aggregate knowledge score ranged between zero and 15. Additionally, items regarding attitude measure were completed to state participants’ level of agreement; disagree (one score), not certain (two scores) or agree (three scores). The aggregate attitudes score ranged between 15 and 45. Cronbach’s alpha coefficients were 0.769 and 0.739 for items regarding knowledge and attitudes, respectively.

Use of Quit with US measures including perceived usefulness of each of the five main pages, satisfaction and confidence in the smartphone app were rated on a five-point scale from one (the lowest) to five (the highest). Participants’ answers were calculated as an average score for each question item. Cronbach’s alpha coefficients were 0.922 and 0.878 for items regarding satisfaction and confidence, respectively.

### 2.9. Sample Size

The sample size calculation was based on a related study evaluating mobile phone text message intervention among university students [43]. This related study was referred to because in 2018, during the study protocol development, no related study regarding smartphone apps for smoking cessation among young adult smokers in Thailand was published. Seven-day point prevalence abstinence rates at 12-week follow-up were predicted as 32.3% for the intervention group vs. 15.9% for the control group. An allocation of 1:1 was employed with 80% desired power level and 5% two-sided significance level. After an attrition rate of 20% was considered, 133 were determined as the sample size for each group, for a total required sample size of 266.

### 2.10. Statistical Analysis

Analyses were computed using Stata 14 Software (StataCorp LP, 2015, College Station, TX, USA). Statistical significance level of <0.05 was applied in a two-tailed test. Fisher’s exact test was employed to compare differences between the Quit with US intervention group and the control group for categorical variables of baseline characteristics, and for the primary smoking abstinence outcome of exhaled CO concentration-level verified 7-day point prevalence. Independent *t*-test was employed to compare differences for continuous variables of baseline characteristics and of all secondary outcomes. The primary outcome was described as risk ratio (RR) and 95% confidence intervals (CIs) and reported as intention-to-treat and then as per protocol principles. In addition, the Generalized Estimating Equation (GEE) was employed to summarize the correlation of repeated smoking abstinence measurements within the same subjects across all follow-ups. This study specified the GEE models using an exchangeable correlation structure and the Poisson distribution with the log link function.

## 3. Results

### 3.1. Recruitment and Baseline Characteristics

**Figure 2:**
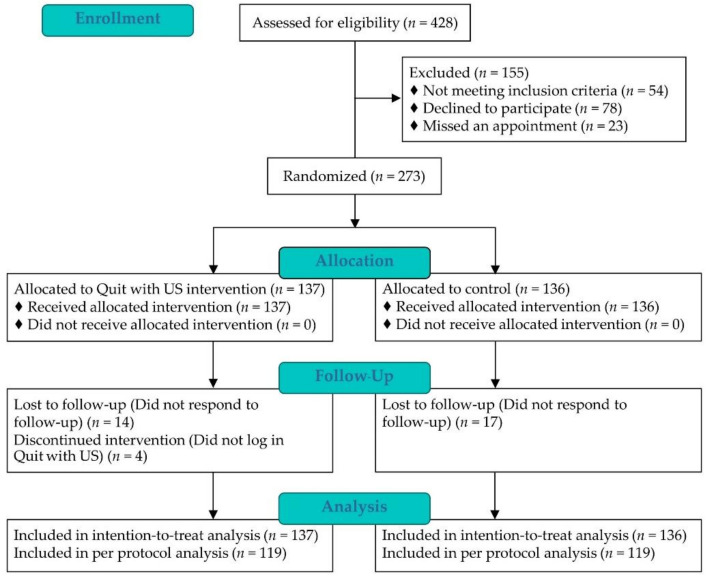
CONSORT flow diagram of Quit with US Trial.

A total of 428 individuals were assessed for eligibility, of whom 155 participants were excluded, resulting in 273 participants randomly assigned: 137 to the Quit with US intervention group and 136 to the control group. The differential of follow-up retention rates at 12 weeks was not observed between the two groups (86.9% (119/137) vs. 87.5% (119/136), *p* = 1.000) (Figure 2).

At baseline, the participants’ mean (standard deviation (SD)) age was 21.06 (1.62) years. Most were males (60.1%, *n* = 164), identified as daily smokers (57.9%, *n* = 158), consumed less than or equal to 10 cigarettes daily (89.4%, *n* = 244), and expressed low level of nicotine dependence as measured by HSI score (86.1%, *n* =235). The Quit with US intervention group and the control group were apparently balanced in baseline characteristics (*p* ≥ 0.05) (Table 1).

No smoking cessation products were provided to any of the participants. In accordance with Thai smoking cessation guidelines, smoking cessation products were offered to 38 participants, 20 in the Quit with US intervention group and 18 in the control group, but all declined and intended to quit smoking by receiving Quit with US integrated with pharmacists’ smoking cessation counseling or receiving only pharmacists’ smoking cessation counseling.

### 3.2. Smoking Abstinence Outcome

**Figure 3:**
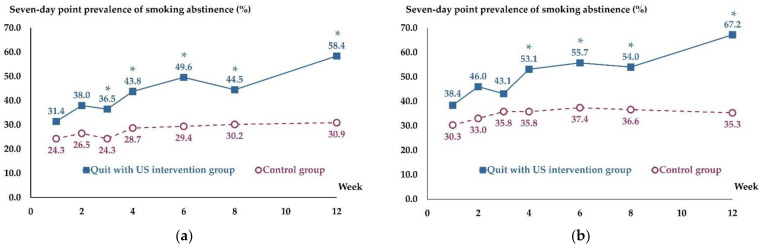
Seven-day point prevalence of smoking abstinence at 1, 2, 3, 4, 6, 8, and 12 weeks follow-up from: (**a**) intention-to-treat analysis; (**b**) per protocol analysis. * *p* < 0.05 was considered statistically significant. Fisher’s exact test was employed to compare differences between the Quit with US intervention group and the control group.

The primary smoking abstinence outcome was intention-to-treat analysis. The number of participants achieving an exhaled CO concentration level verified 7-day point prevalence abstinence at 12-week follow-up significantly increased in the Quit with US intervention group than that in the control group (58.4% (80/137) vs. 30.9% (42/136), RR = 1.89, 95% CI = 1.42 to 2.52, *p* < 0.001) (Table 2 and Figure 3a). The findings remained similar when abstinence rates were assessed per protocol (Table 2 and Figure 3b).

According to the GEE, the number of participants achieving a 7-day point prevalence abstinence across all follow-ups, including the self-report at 1, 2, 3, 4, 6, and 8 weeks, and the exhaled CO concentration level verified at 12 weeks, significantly increased in the Quit with US intervention group from both the intention-to-treat analysis (RR = 1.56, 95% CI = 1.13 to 2.14, *p* = 0.006) and per protocol analysis (RR = 1.54, 95% CI = 1.12 to 2.09, *p* = 0.007).

In addition, when participants were divided in daily (*n* = 158) and nondaily smokers (*n* = 115), the number of participants achieving an exhaled CO concentration-level verified 7-day point prevalence abstinence at 12-week follow-up from both the daily and nondaily smokers significantly increased in the Quit with US intervention group than that in the control group (Table S2).

### 3.3. Secondary Outcomes

Secondary outcome data were collected from participants completing the 12-week follow-up, including 119 participants in each group. The mean (SD) decrease in daily cigarette consumption was significantly greater in the Quit with US intervention group than that in the control group (−4.50 (3.74) vs. −3.28 (3.50), *p* = 0.010). Focusing on nonabstainers unable to quit smoking, their smoking behaviors also improved significantly (Table 3).

The mean (SD) decrease in exhaled CO concentration level was significantly greater in the Quit with US intervention group (−3.60 (3.56) vs. −2.44 (3.83), *p* = 0.016). Furthermore, the mean increase in knowledge and attitudes toward smoking and smoking cessation were also significantly greater in the Quit with US intervention group (Table 3).

### 3.4. Use of Quit with US

In all, 119 participants in the Quit with US intervention group were satisfied with the overall design and the overall content with a mean (SD) score of 4.06 (0.82) and 4.16 (0.80) (scale of 1 to 5), respectively. They also expressed confidence in using the smartphone app with a mean (SD) score of 4.33 (0.74) (scale of 1 to 5) (Table 4, Tables S3 and S4).

## 4. Discussion

This RCT determined the efficacy of Quit with US, a novel smartphone app for smoking cessation, among young adult smokers at 12-week follow-up. Participants in the Quit with US intervention group, using Quit with US integrated with pharmacists’ smoking cessation counseling, achieved significantly greater exhaled CO concentration-level verified 7-day point prevalence abstinence rate than those in the control group, receiving only pharmacists’ smoking cessation counseling. Additionally, participants in the Quit with US intervention group significantly improved in smoking behaviors, exhaled CO concentration level, and knowledge and attitudes toward smoking and smoking cessation. Moreover, participants using Quit with US perceived usefulness, expressed satisfaction, and confidently used the smartphone app.

Regarding the primary smoking abstinence outcome, participants in the Quit with US intervention group achieved almost twice greater smoking abstinence rate than those in the control group. The findings were strengthened by the results from the GEE summarizing the correlation of repeated smoking abstinence measurements within the same subjects across all follow-ups. Remarkably, the smoking abstinence rates of all participants in both groups might have been influenced by the pharmacists’ smoking cessation counseling at baseline and follow-ups. In addition, even when participants in the control group only received pharmacists’ smoking cessation counseling, their smoking abstinence rate was found to be comparable to a related study of smoking cessation services by community pharmacists in Thailand (28.8% of self-reported continuous abstinence for at least 30 days) [13].

The findings might be comparable to the results from related systematic reviews of other smoking cessation approaches with study periods ranging from 6 to 12 months, including community pharmacy personnel interventions for smoking cessation (RR = 2.30, 95% CI = 1.33 to 3.97) [10], telephone counseling for smoking cessation (RR = 1.38, 95% CI = 1.19 to 1.61) [44], and mobile phone text messaging for smoking cessation (RR = 1.54, 95% CI 1.19 to 2.00) [25]. On the contrary, the findings were inconsistent with the results from related studies of smartphone apps for smoking cessation. The systematic reviews of five RCTs measuring smoking abstinence by biochemical validation at six months [25] and the meta-analysis study of eight RCTs measuring smoking abstinence by self-report from the study period of two to six months [45] provided no evidence that smartphone apps could improve the likelihood of smoking cessation. The inconsistency might partly be due to limited RCTs included in these related studies. Therefore, these related studies recommended further studies to examine the effectiveness of smartphone apps. In addition, they suggested that smartphone apps might prove beneficial adjuncts in some populations, such as young adult smokers, due to their low cost, wide availability, and lack of side effects [25,45].

Additionally, the findings were comparable to the results from related RCTs revealing that the abstinence rates significantly increased among participants in the smartphone apps intervention group than those in the control group, ranging from 23.5 to 79.3% vs. 14.0 to 71.1% [17,19]. The variation of these abstinence rates was possibly due to the differences in the measurement of smoking abstinence, the common smoking cessation treatment that all participants received, and the components and contents of smartphone apps. Compared with related RCTs measuring smoking abstinence by the 7-day point prevalence at 12-week follow-up, the significant increase in smoking abstinence rates observed in the present study was comparable to the related RCTs conducted in the US [19] and Japan [17]. The US study revealed that the self-reported abstinence rate significantly increased among participants using a smartphone app based on Acceptance and Commitment Therapy than those using a National Cancer Institute smoking cessation app based on US clinical practice guidelines (23.5 vs. 14.0%, aOR = 1.93, 95% CI = 1.56 to 2.40, *p* < 0.001) [19]. Moreover, the Japanese study revealed that the exhaled CO concentration level (≤10 ppm) verified abstinence rate significantly increased among participants using a smartphone app connected with a CO checker than those using a minimally supportive control app without a CO checker (79.3 vs. 71.1%, *p* = 0.024) [17].

Considering the differences in the common smoking cessation treatments other than smartphone apps provided for all participants in both groups, these might partly have caused the variation in the smoking abstinence rates between the present study and the related studies. In the present study, all participants received pharmacists’ smoking cessation counseling at baseline and follow-ups, while participants in the Japanese study [17] received smoking cessation counseling and pharmacotherapy, and participants in the US study [19] received nothing but smartphone apps. Notably, despite the significant increase in smoking abstinence rate of participants in the Quit with US intervention group observed in the present study, pharmacists’ smoking cessation counseling with and without Quit with US might be insufficient for young adult smokers having a greater likelihood of being heavy cigarette smokers. Focusing on young adult smokers unable to quit smoking in both the Quit with US intervention and control groups, they smoked a greater number of daily cigarettes and reported a greater HSI score (Table 3). The findings suggested that additional treatments, for instance, pharmacotherapy, might be required to assist them in smoking cessation.

In addition, the differences in the components and contents of the smartphone apps might partly have caused the variation in the smoking abstinence rates. These smartphone apps were invented following different approaches. Therefore, the self-instructional smoking cessation materials contained in smartphone apps differed. Quit with US contains illustrations and texts to assist in smoking cessation, while the smartphone app in the Japanese study [17] has animated video lessons. Furthermore, the additional social support contained in smartphone apps differed. The page named Talk with US in the Quit with US app arranges follow-up communication by displaying a list of questions from anonymous users and pharmacists’ answers, while the smartphone app in the Japanese study [17] contains a personalized chatbot for counseling. Notably, considering that Talk with US was not a real-time chat, pharmacists might be able to manage their time away from routine work to answer the queries.

The following potential factors could influence the smoking abstinence rates of participants observed in the present study. First, all participants had an intention of quitting smoking since they participated in the present study. Owing to the inclusion criteria, participants had to be interested in smoking cessation in the next 30 days. This criterion was in line with related RCTs [17,19,24]. Second, all participants received smoking cessation counseling by experienced pharmacists at baseline and follow-ups. For participants in the Quit with US intervention group, combining Quit with US and pharmacists’ smoking cessation counseling might have encouraged mutual support in achieving smoking cessation. Third, participants in the Quit with US intervention group might have received additional support from the page named Talk with US. They were able to anonymously post queries and receive answers from pharmacists throughout the smoking cessation periods. Fourth, Quit with US was invented following the national guidelines for smoking cessation treatment, particularly concentrating on the 5A’s model and self-efficacy theory. The 5A’s model and self-efficacy theory have been implemented in various evidence-based smoking cessation approaches, including health professionals’ counseling [33,34], self-help materials [37], and mobile phone text messaging [38]. Finally, the components and contents of Quit with US were addressed suitably for young adult smokers. The high score of satisfaction and confidence in Quit with US manifested a strong intention to use the smartphone app (Table 4). This might account for young adult smokers’ participation throughout the smartphone app’s invention process: conceptualizing the framework, assessing the smartphone app prototype, and evaluating the preliminary use. Although the findings were inadequate to ascertain the components contributing to users’ success with smoking cessation, Quit with US and Success of US pages received a high score of perceived usefulness (Table 4). These pages were similar in the dynamically tailored content based on users’ input data. Notably, these results might constitute possible means of enhancing user engagement, satisfaction, and confidence in the smartphone app.

Future research on Quit with US is warranted. A national scale RCT covering a study period of at least six months with a wide-ranging group of young adult smokers, including heavy cigarette smokers, should be conducted to confirm the efficacy of Quit with US. In addition, it would be relevant to determine the effects of using only Quit with US on smoking cessation among young adult smokers.

### Limitations

Four limitations should be acknowledged. First, the study period of 12 weeks might have been insufficient to precisely determine that participants could achieve smoking cessation for a longer period. Therefore, future RCTs over a longer study period is warranted to verify the long-term efficacy of Quit with US. Second, blinding was not feasible due to the nature of the study protocol. However, the effect of bias was mitigated using an exhaled CO concentration level of ≤6 ppm to verify 7-day point prevalence abstinence. Third, this study included participants smoking minimally one cigarette within the previous 30 days and excluded participants using other forms of tobacco products. The findings may have limited generalizability among young adults being heavy cigarette smokers and using other forms of tobacco apart from cigarettes. Lastly, participants in this study were restricted to a sample of young adults in northern Thailand. Therefore, the findings may have limited generalizability in other age group populations or among young adults studying in other parts of the country due to their differences in smoking behaviors, smoking cessation behaviors, and smartphone use behaviors.

## 5. Conclusions

This study contributed to the growing evidence that Quit with US integrated with pharmacists’ smoking cessation counseling at baseline and follow-ups significantly enhanced smoking abstinence rates among young adults consuming a few cigarettes daily and expressing a low level of nicotine dependence at 12 weeks. Pharmacists can consider integrating Quit with US with their smoking cessation services to encourage young adult smokers to achieve smoking cessation. However, a future RCT over a more extended study period with a comprehensive group of populations is warranted to verify the efficacy of Quit with US.

## Figures and Tables

**Table 1:** Baseline characteristics of participants in the Quit with US trial.

Characteristic	Quit with US (*n* = 137), *n* (%)	Control (*n* = 136), *n* (%)	Total (*n* = 273), *n* (%)	*p*-Value
**Sex**				
Male	80 (58.4)	84 (61.8)	164 (60.1)	0.622
Female	57 (41.6)	52 (38.2)	109 (39.9)	
**Age, mean (SD), years**	21.20 (1.63)	20.93 (1.60)	21.06 (1.62)	0.179
**Age at cigarette initiation, mean (SD), years**	17.14 (2.29)	16.93 (2.56)	17.04 (2.43)	0.486
**Cigarette smoking frequency**				
Daily smokers	75 (54.7)	83 (61.0)	158 (57.9)	0.327
Nondaily smokers	62 (45.3)	53 (39.0)	115 (42.1)	
**Daily cigarette consumption**				
≤10	123 (89.8)	121 (89.0)	244 (89.4)	0.768
11–20	13 (9.5)	15 (11.0)	28 (10.3)	
21–30	1 (0.7)	0	1 (0.4)	
mean (SD)	5.74 (4.93)	5.87 (4.63)	5.80 (4.77)	0.822
**Time to first cigarette after waking up**				
≤5 min	18 (13.1)	15 (11.0)	33 (12.1)	0.336
6–30 min	14 (10.2)	24 (17.6)	38 (13.9)	
31–60 min	22 (16.1)	18 (13.2)	40 (14.6)	
>60 min	83 (60.6)	79 (58.1)	162 (59.3)	
**HSI score ^1^**				
0–2 (low nicotine dependence)	117 (85.4)	118 (86.8)	235 (86.1)	1.000
3–4 (moderate nicotine dependence)	19 (13.9)	18 (13.2)	37 (13.6)	
5–6 (high nicotine dependence)	1 (0.7)	0	1 (0.4)	
mean (SD)	0.87 (1.22)	0.93 (1.22)	0.90 (1.22)	0.695
**Past year cigarette quit attempt**				
Yes	102 (74.4)	96 (70.6)	198 (72.5)	0.500
No	35 (25.6)	40 (29.4)	75 (27.5)	
**Cigarette quit intention**				
Ready to quit smoking	35 (25.6)	32 (23.5)	67 (24.5)	0.779
In the next 30 days	102 (74.4)	104 (76.5)	206 (75.5)	
**Smartphone operating system**				
iOS	68 (49.6)	74 (54.4)	142 (52.0)	0.468
Android	69 (50.4)	62 (45.6)	131 (48.0)	
**Smartphone use frequency per day**				
≤10 times	13 (9.5)	18 (13.2)	31 (11.4)	0.456
11–20 times	36 (26.3)	42 (30.9)	78 (28.6)	
21–30 times	45 (32.8)	43 (31.6)	88 (32.2)	
≥31 times	43 (31.4)	33 (24.3)	76 (27.8)	
**Smartphone use period per time**				
≤15 min	21 (15.3)	27 (19.8)	48 (17.6)	0.541
16–30 min	45 (32.8)	52 (38.2)	97 (35.5)	
31–45 min	22 (16.1)	20 (14.7)	42 (15.4)	
46–60 min	26 (19.0)	21 (15.4)	47 (17.2)	
≥61 min	23 (16.8)	16 (11.8)	39 (14.3)	
**Previous use of smartphone app for smoking cessation**				
Yes	0 (0)	0 (0)	0 (0)	-
No	137 (100.0)	136 (100.0)	273 (100.0)	
**Exhaled CO concentration, mean (SD), ppm**	9.34 (4.79)	9.48 (4.33)	9.41 (4.56)	0.787
**Scores on knowledge and attitudes**				
Knowledge of smoking and smoking cessation, ^2^ mean (SD)	12.82 (1.48)	12.60 (1.56)	12.71 (1.52)	0.228
Attitudes toward smoking and smoking cessation, ^3^ mean (SD)	39.74 (2.99)	39.18 (3.28)	39.46 (3.14)	0.141

HIS, Heaviness of Smoking Index; CO, carbon monoxide; ppm, parts per million; ^1^ The scores ranged between 0 and 6, with high scores conveying high nicotine dependence. ^2^ The scores ranged between 0 and 15, with high scores conveying better knowledge on smoking and smoking cessation. ^3^ The scores ranged between 15 and 45, with high scores conveying positive attitudes toward smoking and smoking cessation.

**Table 2:** Primary outcome in the Quit with US trial.

Exhaled CO Concentration Level Verified 7-Day Point Prevalence Abstinence ^1^	Quit with US, *n* (%)	Control, *n* (%)	RR (95% CI)	*p*-Value
**Intention-to-treat analysis**	(*n* = 137)	(*n* = 136)		
Abstainers	80 (58.4)	42 (30.9)	1.89 (1.42 to 2.52)	<0.001
Nonabstainers	57 (41.6)	94 (69.1)	1.00	
**Per protocol analysis**	(*n* = 119)	(*n* = 119)		
Abstainers	80 (67.2)	42 (35.3)	1.90 (1.45 to 2.50)	<0.001
Nonabstainers	39 (32.8)	77 (64.7)	1.00	

RR, risk ratio; CI, confidence intervals; ^1^ Measured by a self-report of continuous abstinence from smoking in the previous 7 consecutive days plus an exhaled CO concentration level of ≤6 ppm.

**Table 3:** Secondary outcomes in the Quit with US trial.

Outcome Variable	Quit with US (*n* = 119), Mean (SD)			Control (*n* = 119), Mean (SD)			*p*-Value ^1^
	Baseline	Follow-Up	Change (Follow-Up -Baseline)	Baseline	Follow-Up	Change (Follow-Up -Baseline)	
**Smoking behaviors ^2^**							
Daily cigarette consumption	5.71 (4.77)	1.21 (2.38)	−4.50 (3.74)	5.94 (4.54)	2.66 (3.20)	−3.28 (3.50)	0.010
HSI score ^3^	0.86 (1.20)	0.13 (0.53)	−0.73 (1.12)	0.92 (1.22)	0.34 (0.76)	−0.59 (0.94)	0.288
**Smoking behaviors of nonabstainers**		(*n* = 39)			(*n* = 77)		
Daily cigarette consumption	9.20 (5.61)	3.69 (2.88)	−5.51 (4.60)	7.08 (4.67)	4.10 (3.15)	−2.97 (3.49)	0.004
HSI score ^3^	1.56 (1.33)	0.38 (0.88)	−1.18 (1.27)	1.22 (1.27)	0.52 (0.90)	−0.70 (0.95)	0.043
**Exhaled CO concentration level** **(ppm)**	9.43 (4.85)	5.82 (3.78)	−3.60 (3.56)	9.48 (4.34)	7.04 (3.98)	−2.44 (3.83)	0.016
**Scores on knowledge and attitudes**							
Knowledge of smoking and smoking cessation ^4^	12.86 (1.42)	13.94 (1.10)	1.08 (1.38)	12.63 (1.53)	13.22 (1.38)	0.59 (1.40)	0.006
Attitudes toward smoking and smoking cessation ^5^	39.76 (3.02)	41.92 (2.54)	2.16 (2.57)	39.34 (3.35)	40.34 (2.84)	0.99 (2.80)	0.001

HIS, Heaviness of Smoking Index; CO, carbon monoxide; ppm, parts per million; ^1^ Independent *t*-test was employed to compare differences in secondary outcome change between the Quit with US intervention group and the control group. ^2^ Participants achieving biochemically verified 7-day point prevalence abstinence recorded 0 daily cigarette consumption and 0 HSI score. ^3^ The scores ranged between 0 and 6, with high scores conveying high nicotine dependence. ^4^ The scores ranged between 0 and 15, with high scores conveying better knowledge on smoking and smoking cessation. ^5^ The scores ranged between 15 and 45, with high scores conveying positive attitudes toward smoking and smoking cessation.

**Table 4:** Use of Quit with US of 119 participants in the intervention group.

Variable	Mean (SD)
**Frequency of using the smartphone app per day, *n* (%)**	
1 time	87 (73.1)
≥2 times	32 (26.9)
**Period of using the smartphone app per time, *n* (%)**	
≤5 min	66 (55.5)
6–10 min	49 (41.2)
11–15 min	4 (3.4)
**Perceived usefulness of each main page ^1^**	
Suggested by US	3.91 (0.89)
Talk with US	3.40 (1.01)
Quit with US	4.17 (0.83)
Let US Help	3.61 (0.99)
Success of US	4.10 (0.97)
**Satisfaction with the overall design ^1^**	4.06 (0.82)
**Satisfaction with the overall content ^1^**	4.16 (0.80)
**Confidence in the overall use ^1^**	4.33 (0.74)

^1^ The mean scores ranged between 1 and 5, with high scores conveying better perceived usefulness, satisfaction or confidence.

## Data Availability

The data presented in this study are available from the corresponding author on reasonable request.

## Supplementary Materials

The following supporting information can be downloaded at: https://www.mdpi.com/article/10.3390/ijerph19148265/s1, Table S1: Brief manual for pharmacists’ smoking cessation counseling, Table S2: Primary outcome in the Quit with US trial by separating daily and nondaily smokers, Table S3: Satisfaction in Quit with US of 119 participants in the intervention group, Table S4: Confidence in Quit with US of 119 participants in the intervention group.
