# Supplementary material for: Smartphone Application for Smoking Cessation (Quit with US): A Randomized Controlled Trial among Young Adult Light Smokers in Thailand
Source: Int J Environ Res Public Health. 2022 Jul 6;19(14):8265. doi: 10.3390/ijerph19148265 (PMC9321212; doi:10.3390/ijerph19148265)
Supplement: Supplementary file 1 [file ijerph-19-08265-s001.zip › ijerph-1754967-Table S1 for Revised Manuscript Round 1 (21-6-2565).pdf]

Table S1. Brief manual for pharmacists' smoking cessation counseling

5 A's	Action
***Ask* participants about cigarette smoking behaviors.**	Ask participants about cigarette and tobacco products use, for instance, the following.<br>• "When did you start smoking?"<br>• "How often did you smoke?"<br>• "What was your reason for smoking your first cigarette?"
***Advise* participants to quit smoking.**	Recommend participants to quit in a clear, strong, and personalized manner, for instance, the following.<br>• "It is important that you quit smoking now, and I can help you."<br>• "Quitting smoking is the most important thing you can do to protect your health."<br>• "You can save money by quitting smoking."
***Assess* readiness to make a quit attempt and nicotine dependence level.**	Assess readiness to make a quit attempt, for instance, the following.<br>• "Are you ready to quit smoking now?"<br>• "Do you think you have a chance of quitting successfully?"<br>Assess nicotine dependence level using the Heaviness of Smoking Index (HSI) by asking these two questions.<br>• "How many cigarettes do you smoke daily?"<br>- 1-10 cigarettes (0 score)<br>- 11-20 cigarettes (1 score)<br>- 21-30 cigarettes (2 scores)<br>- ≥31 cigarettes (3 scores)<br>• "How soon after waking up do you smoke your first cigarette?"<br>- Within 5 minutes (3 scores)<br>- 6-30 minutes (2 scores)<br>- 31-60 minutes (1 score)<br>- ≥61 minutes (0 score)<br>The HSI summed scores are separated in 3 levels of nicotine dependence.<br>• Low nicotine dependence (0-2 scores)<br>• Moderate nicotine dependence (3-4 scores)<br>• High nicotine dependence (5-6 scores)<br>Assess causes of cigarette dependence, for instance, the following.<br>• "When do you usually smoke a cigarette?"<br>• "What do you usually do while cigarette smoking?"<br>• "What do you feel when you smoke a cigarette?"
***Assist* participants with a quit plan.**	Participants who are ready to quit smoking are assisted in smoking cessation using the STAR technique, for instance, the following.<br>• "Set a target quit date." "The target quit date should ideally be within 2 weeks."<br>• "Tell your families, friends, and coworkers about your decision to quit, and ask for their understanding and support."<br>• "Anticipate challenges to your upcoming quit attempt, especially during the critical first few weeks, including nicotine withdrawal symptoms, using the 5D's."<br>- Delay: "Delay until the craving to smoke has passed."<br>- Deep breath: "When you feel the urge to smoke, try breathe deeply."<br>- Drink water: "Drinking plenty of water." "Sipping water when urged to smoke a cigarette."<br>- Do something else: "Distract yourself from thoughts of smoking by doing something else."<br>- Destination: "Keep reminding yourself that you would like to quit successfully."<br>• "Remove all tobacco-related products from your home and make it smoke free." "Stay away from places where you used to smoke."

Table S1. (Continued)

5 A's	Action
	Participants who were not ready to quit smoking were assisted in smoking cessation using the 5R's.<br>• Relevance: Indicate how smoking is relevant to personal health problems.<br>• Risks: Identify potential negative consequences of smoking that are relevant to personal health problems.<br>• Rewards: Emphasize personal benefits of quitting smoking.<br>• Roadblocks: Determine barriers to quitting and devise a solution.<br>• Repetition: Repeat the motivation to quit at every visit.
***Arrange* for follow-up**	Arrange for follow-up at 1, 2, 3, 4, 6, 8 and 12 weeks.<br>• Identify encountered problems.<br>• Anticipate challenges in the immediate future.<br>For participants who can quit smoking<br>• Congratulate and affirm decision to quit.<br>• Offer ongoing encouragement after quitting.<br>For participants who cannot quit smoking or relapse.<br>• Enhance motivation to quit using the 5R's.<br>• Explore reasons for relapse, resolve and prevent for future lessons.<br>• Set a new target quit date.
