# Supplementary material for: Smartphone Application for Smoking Cessation (Quit with US): A Randomized Controlled Trial among Young Adult Light Smokers in Thailand
Source: Int J Environ Res Public Health. 2022 Jul 6;19(14):8265. doi: 10.3390/ijerph19148265 (PMC9321212; doi:10.3390/ijerph19148265)
Supplement: Supplementary file 1 [file ijerph-19-08265-s001.zip › ijerph-1754967-Table S2 for Revised Manuscript Round 1 (21-6-2565).pdf]

Table S2. Primary outcome in the Quit with US trial by separating daily and nondaily smokers

**Exhaled CO concentration level verified 7-day point prevalence abstinence [1]**	**Daily smokers**				**Nondaily smokers**			
	**Quit with US, *n* (%)**	**Control, *n* (%)**	**RR (95% CI)**	***p***	**Quit with US, *n* (%)**	**Control, *n* (%)**	**RR (95% CI)**	***p***
**Intention-to-treat analysis**	(*n* = 75)	(*n* = 83)			(*n* = 62)	(*n* = 53)		
Abstainers	28 (37.3)	8 (9.6)	3.87 (1.88 to 7.96)	<0.001	52 (83.9)	34 (64.2)	1.31 (1.04 to 1.64)	0.018
Nonabstainers	47 (62.7)	75 (90.4)	1.00		10 (16.1)	19 (35.8)	1.00	
**Per protocol analysis**	(*n* = 64)	(*n* = 74)			(*n* = 55)	(*n* = 45)		
Abstainers	28 (43.8)	8 (10.8)	4.05 (1.99 to 8.24)	<0.001	52 (94.6)	34 (75.6)	1.25 (1.05 to 1.49)	0.009
Nonabstainers	36 (56.2)	66 (89.2)	1.00		3 (5.4)	11 (24.4)	1.00	

RR, risk ratio; CI, confidence intervals; [1] Measured by a self-report of continuous abstinence from smoking in the previous 7 consecutive days plus an exhaled CO concentration level of ≤6 ppm.
