# Supplementary material for: Smartphone Application for Smoking Cessation (Quit with US): A Randomized Controlled Trial among Young Adult Light Smokers in Thailand
Source: Int J Environ Res Public Health. 2022 Jul 6;19(14):8265. doi: 10.3390/ijerph19148265 (PMC9321212; doi:10.3390/ijerph19148265)
Supplement: Supplementary file 1 [file ijerph-19-08265-s001.zip › ijerph-1754967-Table S3 for Revised Manuscript Round 1 (21-6-2565).pdf]

**Table S3.** Satisfaction in Quit with US of 119 participants in the intervention group

| Variable | Quit with US, mean (SD) [1] |
|---|---|
| **Satisfaction with the overall design** | **4.06 (0.82)** |
| 1. Font styles and sizes were appropriate and obvious. | 4.33 (0.64) |
| 2. Menu icons could convey a clear understanding of their functions. | 4.28 (0.70) |
| 3. Illustrations and graphics were attractive, interesting, and luminous. | 4.26 (0.69) |
| 4. Component colors on the screen were appropriate and attractive. | 4.23 (0.62) |
| 5. The smartphone app was user-friendly. | 4.13 (0.85) |
| 6. The position arrangement of the components on the screen was appropriate and attractive. | 4.08 (0.68) |
| 7. Number of menu options were appropriate for use. | 4.05 (0.82) |
| 8. Buttons were appropriately arranged and user-friendly. | 4.03 (0.87) |
| 9. The smartphone app runs smoothly without any technical glitch. | 3.17 (0.89) |
| **Satisfaction with the overall content** | **4.16 (0.80)** |
| 1. Displayed presentation of records on users' progress in smoking cessation. | 4.35 (0.72) |
| 2. Advice on smoking cessation methods. | 4.26 (0.78) |
| 3. Self-setting goals in quitting smoking. | 4.24 (0.67) |
| 4. Displayed encouraging messages about smoking cessation. | 4.22 (0.81) |
| 5. Calculated money saved from smoking cessation. | 4.19 (0.90) |
| 6. Information about benefits of smoking cessation to the body. | 4.13 (0.82) |
| 7. Self-set an initial date for smoking cessation. | 4.13 (0.79) |
| 8. Suggested coping methods when having symptoms after smoking cessation. | 4.11 (0.72) |
| 9. Information about drawbacks of smoking to the body. | 4.09 (0.87) |
| 10. Suggested coping methods with a desire to smoke or unintentional smoking. | 4.08 (0.78) |
| 11. A list of questions and answers provided by pharmacists. | 3.95 (0.86) |

[1] The mean scores ranged between 1 and 5, with high scores conveying better satisfaction.
