# Supplementary material for: Smartphone Application for Smoking Cessation (Quit with US): A Randomized Controlled Trial among Young Adult Light Smokers in Thailand
Source: Int J Environ Res Public Health. 2022 Jul 6;19(14):8265. doi: 10.3390/ijerph19148265 (PMC9321212; doi:10.3390/ijerph19148265)
Supplement: Supplementary file 1 [file ijerph-19-08265-s001.zip › ijerph-1754967-Table S4 for Revised Manuscript Round 1 (21-6-2565).pdf]

Table S4 Confidence in Quit with US of 119 participants in the intervention group

Variable	Quit with US, mean (SD) [1]
**Confidence in the overall use**	**4.33 (0.74)**
1. This smartphone app was considered a safe smoking cessation method.	4.55 (0.63)
2. This smartphone app provided accurate information about smoking cessation.	4.55 (0.55)
3. Using this smartphone app as a supplement to other smoking cessation methods could promote successful smoking cessation.	4.54 (0.61)
4. I would suggest those who desire to quit smoking to use this smartphone app.	4.48 (0.66)
5. This smartphone app enabled me to receive suggestions and assistance at my convenience.	4.25 (0.74)
6. This smartphone app was considered a financially favorable method when compared with other smoking cessation methods.	4.17 (0.84)
7. This smartphone app made me feel confident of my ability to successfully quit smoking.	4.04 (0.78)
8. I was confident that this smartphone app would assist me to successfully quit smoking.	4.02 (0.81)

[1] The mean scores ranged between 1 and 5, with high scores conveying better confidence.
